# The Potential Role of Iodine-123 *Meta*iodobenzylguanidine Imaging for Identifying Sustained Ventricular Tachycardia in Patients with Cardiomyopathy

**DOI:** 10.1007/s11886-013-0359-1

**Published:** 2013-03-29

**Authors:** Thomas Klein, Vasken Dilsizian, Qi Cao, Wengen Chen, Timm-Michael Dickfeld

**Affiliations:** 1 Maryland Arrhythmia and Cardiology Imaging Group (MACIG), Department of Medicine, Division of Cardiology, University of Maryland Medical Center, 22 South Greene St, Baltimore, MD 21201 USA; 2 Department of Diagnostic Radiology and Nuclear Medicine, University of Maryland Medical Center, 22 South Greene Street, Baltimore, MD 21201 USA

**Keywords:** Cardiac *m*IBG, Cardiomyopathy, Ventricular tachycardia, Cardiac arrhythmia, Heart failure, Ablation therapy, Cardiomyopathy

## Abstract

Implantable cardioverter-defibrillators (ICDs) significantly reduce mortality in patients with depressed left ventricular ejection fraction (LVEF) and heart failure (HF). However, shortcomings of LVEF to accurately identify those at greatest risk of ventricular tachyarrhythmias have led to the pursuit of alternative means to refine qualification criteria for ICD implantation. It is well established that imaging the cardiac nervous system with^123^I *meta*-iodobenzylguanidine (^123^I-*m*IBG) provides incremental prognostic value in patients with HF beyond LVEF. Whether ^123^I-*m*IBG will also play an important role for identifying and/or predicting sustained ventricular tachyarrhythmias in patients with cardiomyopathy and determining those who may benefit from ICD implantation is currently under investigation. Novel imaging approaches that pinpoint the site of ventricular arrhythmias and guide ventricular tachycardia ablation are presented.

## Introduction

Sudden cardiac death is a significant public health issue, affecting 150,000-450,000 patients in the United States each year [1, 2]. Clinical trial data has shown that implantable cardioverter-defibrillators (ICDs) significantly reduce mortality in patients with depressed left ventricular ejection fraction (LVEF) and heart failure (HF), whereas antiarrhythmic medication has not been shown to have the same benefits compared to placebo [3, 4]. Identifying patients at risk for developing ventricular tachyarrhythmias who will benefit from ICD placement for primary prevention has been the objective of a multitude of large clinical trials over the past two decades. The focus has been on patients with structural heart disease, and the most valuable discriminator currently in use is LVEF. However, the majority of patients who suffer SCD do not have characteristics that would qualify them for an ICD for primary prevention [5, 6]. Furthermore, in a large randomized primary prevention ICD implantation trial, over two thirds of patients who received ICDs with depressed LVEF never received ICD therapy during a follow-up period of almost 2 years [7]. These shortcomings of LVEF to accurately identify those at greatest risk of ventricular arrhythmias (VTA) have led to the pursuit of alternative means to refine implantation qualification criteria for ICDs.

Signal-averaged electrocardiogram (ECG), microvolt T-wave alternans, electrophysiologic testing, serum markers (including brain natriuretic peptide), and autonomic function evaluation (including heart rate variability (HRV), baroreflex sensitivity, heart rate turbulence, and deceleration capacity of heart rate) have all been studied, and have produced variable results [8]. More recently, imaging the cardiac nervous system has proven to have incremental prognostic value in patients with HF beyond LVEF and B-type natriuretic peptide (BNP).This article will summarize the data on imaging cardiac innervations with ^123^I *meta*-iodobenzylguanidine (^123^I-*m*IBG) SPECT and its potential role in predicting the risk of VTA and describe novel imaging approaches to identify the site of VTA to guide ventricular tachycardia (VT) ablation.

## Imaging the Cardiac Sympathetic Nervous System

^123^I-*m*IBG has been the most common radiotracer studied for imaging cardiac innervation. It was first introduced in 1979 for imaging the adrenal medulla, and for the heart shortly thereafter [9]. ^123^I-*m*IBG is chemically modified from guanethidine, which is an analogue of the endogenous neurotransmitter norepinephrine, and uses the same uptake and storage mechanisms as norepinephrine. Two mechanisms of ^123^I-*m*IBG uptake from the synaptic cleft have been identified, one being neuronal and the other being non-neuronal, of which the former predominates in the human heart. This is evidenced by the fact that uptake is absent in enervated transplanted hearts [10]. Unlike norepinephrine, ^123^I-*m*IBG is not metabolized by monoamine oxidase or catechol-O-methyltransferase, and does not interact with postsynaptic receptors. Thus, cardiac ^123^I-*m*IBG reflects uptake in only presynaptic sympathetic fibers in the myocardium [11].

^123^I-*m*IBG cardiac images are usually acquired in the anterior planar view 5–40 minutes (early) and again 3–4 hours (delayed) after the injection of the radiotracer. From these planar images, the heart-to-mediastinal ratio (H/M) is calculated by dividing the mean counts per pixel from a cardiac region of interest by the mean counts per pixel from an area in the upper mediastinum. Delayed H/M ratio derived from the anterior planar view has been widely used to predict patient outcome and monitor response to medical treatment. In addition, ^123^I-*m*IBG washout rate (WR) is calculated by comparing early and delayed ^123^I-*m*IBG activities in the heart, reflecting the retention or turnover of ^123^I-*m*IBG in neurons. After each planar acquisition, a SPECT acquisition is performed, and images are analyzed in conventional orthogonal planes (short axis, vertical long axis, and horizontal long axis).

The reproducibility and inter-observer variability of ^123^I-*m*IBG in HF patients at single centers has been found to be acceptable and highly reproducible [12]. However, inter-institutional variations exist which have been attributed to the use of different collimators, image acquisition parameters, injected doses, region of interest settings, ^123^I-*m*IBG labeling methods, disease status, and ^123^I-*m*IBG isotopes[13]. Also, some patterns across patient populations have been noted. For example, in healthy subjects, inferior wall uptake of ^123^I-*m*IBG may decrease with age, especially in men [14]. Given the inter-institutional variations, a standardized protocol was recently proposed by the Cardiovascular Committee and the European Council of Nuclear Cardiology [11].

## The Clinical Utility of^123^I-*m*IBG in HF

The autonomic nervous system is known to play a key role in the pathophysiology of HF. Neurohormonal feedback provides a means for compensation in the early stages of HF; however, as the disease progresses, chronic sympathetic output leads to detrimental effects, including interstitial fibrosis and left ventricular remodeling. Chronically elevated norepinephrine levels have been linked to an increased risk of mortality in HF [15]. Additionally, pharmacologic blockade of the sympathetic input to the heart decreases mortality in these patients [16, 17].

Multiple clinical trials have evaluated ^123^I-*m*IBGin HF patients. Patients with HF exhibit decreased cardiac uptake of ^123^I-*m*IBG (decreased H/M), and earlier release of ^123^I-*m*IBG from early to delayed imaging due to compromised neuronal integrity (increased WR). An inverse relationship has been shown between severity of HF classification and H/M ^123^I-*m*IBG ratio. Retrospective and single center studies over the past two decades have demonstrated that ^123^I-*m*IBGuptake predicts the risk of cardiac death cardiomyopathy (Table 1) [18–25]. Preliminary data also suggest salutary effects of medical therapy on cardiac ^123^I-*m*IBG uptake and its relation to clinical outcomes [19, 26]. Several small series have demonstrated improvement in sympathetic innervation, as assessed with ^123^I-*m*IBGscintigraphy, resulting from cardiac resynchronization therapy and left ventricular assist device therapy, which paralleled multiple clinical parameters [27–29]. Conversely, non-responders to CRT did not demonstrate the same improvements in sympathetic innervation [30, 31].

**Table 1:** Relevant Studies studies on *m*IBG Scintigraphy scintigraphy for Prognosis prognosis in HF

Year	Author	n=	Etiology (% ICM)	Mean baseline LVEF (%)	NYHA Class	Mean Follow-up (months)	Result
1992	Merlet, et al. [18]	90	27	22	II-IV	11	H/M was more valuable in predicting survival than x-ray cardiothoracic ratio, echocardio-graphic end-diastolic diameter and radionuclide LVEF
1998	Nakata, et al. [35]	414	32	49	1.6 (mean)	22	Late H/M, early H/M, use of nitrates, and LVEF were all predictive of cardiac death, but late H/M was the most powerful predictor
1999	Cohen-Solal, et al. [20]	93	26	25	2.6 (mean)	10	In patients with chronic HF, late H/M and peak oxygen consumption were predictive of death or heart transplantation, but only peak VO2 was significant by multivariate analysis
1999	Merlet, et al. [24]	112	0	21	II-IV	27	Of several variables tested, only late H/M and LVEF were predictive of mortality in idiopathic nonischemic cardiomyopathy
2001	Imamura, et al. [23]	171	56	27	1.9 (mean)	27	Elevated WR was an independent predictor of cardiac death; elevated WR and BNP predicted progressive HF
2001	Ogita, et al. [25]	79	57	29	1.8 (mean)	31	WR ≥ 27 % predicted SCD, HF death, and worsening HF
2002	Gerson, et al. [26]	22	0	25	II-IV	7.2	Patients with abnormal baseline ^123^I-*m*IBG uptake demonstrated improvement in ^123^I-*m*IBG uptake with carvedilol treatment, which correlated with improvement in LVEF.
2003	Kasama, et al. [36]	30	0	33	2.8 (mean)	6	Spironolactone decreased total defect score, and WR and increased H/M in HF patients to placebo. These changes correlated with improvement in LVEF, LV end-diastolic volume, and NYHA class.
2003	Yamada, et al. [21]	65	63	28	2.1 (mean)	34	In multivariate analysis, only WR (not H/M or HRV) was predictive of cardiac events
2005	Nakata, et al. [19]	88	27	27	2.6 (mean)	43	Less severe ^123^I-*m*IBG defect correlated with improved treatment effect from beta-blockers and/or ACE inhibitors (Mortality reduction from 36 % to 12 % in those with H/M ≥ 1.53, from 53 % to 37 % in those with H/M < 1.53)
2008	Agostini, et al. [22]	290	42	32	2.5 (mean)	24	In this retrospective analysis, decreased H/M LVEF were both predictive of major cardiac events
2008	Kasama, et al.	208	42	32	2.6 (mean)	53	Patients underwent serial ^123^I-*m*IBG imaging, and ∆ WR was shown to be an incremental predictor of cardiac death and sudden death
2010	Jacobson, et al. [34••]	961	66	27	2.16 (mean)	17	Late H/M, in addition to LVEF, BNP, and NYHA class was an independent predictor of HF progression, arrhythmic events, and cardiac death.

A meta-analysis of 18 small trials including 1755 patients was published in 2008, and demonstrated that decreased H/M and elevated WR portends a worse prognosis, with increased risk of cardiac death and cardiac events [32]. Based on the aforementioned data and similar results in previously published trials, ^123^I-*m*IBG has gained clinical use in Europe and Japan in HF patients and for cardiac transplantation candidacy since the 1990s. In the U.S., however, ^123^I-*m*IBG has not yet received Food and Drug Administration (FDA) approval for cardiac application [33].

AdreView Myocardial Imaging for Risk Evaluation in Heart Failure (ADMIRE-HF) prospective, multinational, multicenter, open-label study which enrolled 961 patients with LVEF ≤ 35 % and New York Heart Association (NYHA) functional class II-III was recently completed [34••]. Patients underwent both ^123^I-*m*IBGand myocardial perfusion imaging and were followed for two years. The subgroup of patients with ^123^I-*m*IBGH/M < 1.6 had a 2-year cardiac event rate (progression of NYHA class, potentially life threatening arrhythmias, or cardiac death) of 37 %, while those with H/M ≥ 1.6 had a 2-year event rate of 15 %. Each of the three components of the primary outcome were also significantly reduced in patients with H/M ≥ 1.6 (composite primary outcome: HR = 0.4, p < 0.001; HF progression: HR = 0.49, p = 0.002; life-threatening arrhythmia: HR = 0.37, p = 0.02; cardiac death: HR = 0.14, p = 0.006). LVEF, BNP, NYHA class, and H/M were significantly predictive of event occurrence in multivariate analysis. Although WR was predictive in univariate analysis, it did not remain so in multivariate analysis.

## ^123^I-*m*IBG for Assessment of Ventricular Arrhythmia Risk

Activation of sympathetic nervous system is an important factor in the pathophysiology of ventricular tachyarrhythmias. All three known mechanisms of ventricular tachyarrhythmias, including enhanced automaticity, triggered automaticity, and reentrance, can be potentiated by the sympathetic nervous system. Sympathetic neuronal innervation has been shown to be denser in those with ventricular arrhythmias than in those without [37]. It is theorized that denervated but viable myocardium may demonstrate an exaggerated response to circulating catecholamines [38].

It was observed over two decades ago that abnormal ^123^I-*m*IBGuptake was present after myocardial infarction (MI) and correlated with ventricular ectopy [39] and with inducible ventricular tachyarrhythmias during invasive electrophysiologic testing [40], as well as in patients without coronary artery disease but with spontaneous ventricular tachyarrhythmias [41]. These small series sparked a long pursuit to uncover the predictive value of ^123^I-*m*IBGfor ventricular tachyarrhythmias, to refine the utilization of ICDs, and to determine a possible clinical role for cardiac ^123^I-*m*IBGtesting. Multiple studies have evaluated the value of ^123^I-*m*IBGimaging in diverse groups of patients to predict the risk of ventricular tachyarrhythmias, sudden cardiac death, and ICD discharges (Table 2) [42–51].

**Table 2:** Relevant studies on *m*IBG scintigraphy and ventricular tachyarrhythmias

Year	Author	n=	Patient Population	Mean baseline LVEF (%)	Mean Follow-up (months)	Result
1991	McGhie, et al. [39]	27	Post-MI	-	-	Higher total defect score on ^123^I-*m*IBG correlated with the presence of VT and ventricular ectopy within 10 days post-MI
2001	Daliento, et al. [57]	22	Post-surgical correction of tetralogy of Fallot	-	-	Those with VTA on 24-hour Holter monitoring had significantly reduced ^123^I-*m*IBG uptake and increased WR than those without.
2003	Arora, et al. [42]	17	Prior ICD discharges	39	-	10 patients with a history of ICD discharges had significantly lower H/M and higher WR, as well as reduced values for several HRV parameters, than 7 patients without prior ICD discharges
2003	Terai, et al. [47]	44	HCM	59	-	Mean WR was significantly higher in 15 patients with VTA on 24-hour Holter monitor than in 29 without.
2006	Paul et al. [43]	20	Idiopathic VTA with structurally normal hearts	72	86	18 recurrent episodes occurred in 4 patients with abnormal ^123^I-*m*IBG uptake, whereas only 2 episodes occurred in 1 patient with normal ^123^I-*m*IBG uptake.
2007	Kioka, et al. [52]	97	CHF (53 % ICM, mean NYHA class 2.1)	29	65	Early and late H/M and WR were all predictive of SCD
2008	Bax, et al. [50]	50	CHF (62 % ICM)	32	-	Patients underwent ^123^I-*m*IBG prior to EP testing. 4-hour ^123^I-*m*IBG TDS ≥ 37 predicted EP positivity.
2009	Akutsu, et al. [44]	86	Prior VTA	59	132	H/M ≤ 2.8 predicted recurrence of VTA (HR 3.6 [95 % confidence interval, 1.4-9.2, P = 0.007]).
2009	Koutelou, et al. [53]	25	Compensated CHF (NYHA class I-II) and recent ICD implantation.	36	32	WR, in addition to HRV and baroreflex sensitivity, predicted ICD discharges
2010	Boogers, et al. [54••]	116	HF	28	23	^123^I-*m*IBG SPECT was performed before ICD implantation. Those with late ^123^I-*m*IBG SPECT defect score > 26 had a greater risk of appropriate ICD therapy (52 % vs. 5 %, p < 0.01).
2010	Nishisato, et al. [45]	60	Diverse group of patients undergoing ICD implantation	49	29	At the time of ICD implant, ^123^I-*m*IBG planar and technetium SPECT perfusion imaging were performed. H/M ≤ 1.9 with summed perfusion score ≥ 12 independently predicted elevated risk of ICD discharge.
2011	Paul, et al. [46]	42	Arrhythmogenic right ventricular cardiomyopathy	-	143	Patients underwent ^123^I-*m*IBG SPECT, and 59 % were found to have abnormal uptake. Abnormal uptake predicted future VTA.
2011	Miranda, et al. [49]	26	Chagas cardiomyopathy with and without VTA	53	-	Compared to patients without of VTA on 24-hour Holter monitoring, those with VTA had an increased ^123^I-*m*IBG defect score.
2012	Kasama, et al. [51]	56	Dilated cardiomyopathy	31	54	Late potentials and ^123^I-*m*IBG imaging was performed. Those with late potential positivity and WR > 50 % had an elevated risk of SCD.
2012	Marshall et al. [48]	27	HF patients receiving an ICD for primary prevention	24	16	Low H/M and high total ^123^I-*m*IBG SPECT defect score at the time of ICD implantation predicts future ICD discharges

### HF Patients.

Prospective observational studies have shown that^123^I-*m*IBG may predict the risk of ICD discharges in patients with mild-to-moderate HF. In 97 patients with LVEF < 40 % and an average NYHA functional class II who underwent cardiac ^123^I-*m*IBG imaging, both WR and early and late H/M were predictive of sudden cardiac death [52]. Over a mean follow-up of 65 months, the prevalence of sudden cardiac death was significantly higher in patients with ^123^I-*m*IBG WR ≥ 27 % compared to those with WR < 27 %; 25%and 4 %, respectively. In another series of patients with NYHA class I-II HF and recent ICD implantation, ^123^I-*m*IBG WR, in addition to baroreflex sensitivity and heart rate variability, was found to correlate directly with the incidence of ICD firings [53].

The largest prospective, multicenter study to date designed to examine the predictive value of ^123^I-*m*IBGscintigraphy for predicting ICD implantation and discharge in HF patients was published in 2010. Among 116 patients (mean LVEF = 28 %, 96 % ischemic etiology, mean NYHA class = 2.9) who underwent ^123^I-*m*IBGcardiac imaging prior to ICD implantation, 52 % of patients with large ^123^I-*m*IBG defects (summed score > 26) received appropriate ICD therapy (primary endpoint) during a mean follow up period of 23 months versus only 5 % of patients with a smaller ^123^I-*m*IBG defect (p < 0.01). Moreover, 57 % of those with a large ^123^I-*m*IBG defect experienced the secondary endpoint of appropriate ICD discharge or cardiac death versus only 10 % of those with smaller defects (p < 0.01) [54••].

### Non-HF Patients.

Populations known to be at risk for ventricular tachyarrhythmias but without a history of HF have also been evaluated for cardiac ^123^I-*m*IBG abnormalities. In patients with Brugada syndrome, 47 % were found to have regional ^123^I-*m*IBG defects, most commonly in the inferior and septal regions [55]. Among patients with long QT syndrome, 61 % were found to have regional ^123^I-*m*IBG defects, most commonly in the anteroseptal region. No difference in ^123^I-*m*IBG uptake pattern was noted between different long QT syndrome subtypes, between those with corrected QT (QTc) > 500 ms vs. those with QTc < 500 ms, or between those suffering from cardiac arrest or syncope [56]. Other groups for which ^123^I-*m*IBG imaging abnormalities may predict the risk for ventricular tachyarrhythmias are those with idiopathic ventricular fibrillation [43, 44], arrhythmogenic right ventricular dysplasia [46],hypertrophic cardiomyopathy [47],Chagas cardiomyopathy [49], and after surgical correction of tetralogy of Fallot [57].

## Integration of 3-Dimensional Scar Models from ^123^I-*m*IBGNeuro-Cardiac Imaging to Guide Ventricular Tachycardia Ablation

The localized information of abnormal ^123^I-*m*IBG uptake pattern in the heart has raised the possibility that regional inhomogeneities of innervation may be related to ventricular arrhythmias and could provide guidance for VT ablations. Magnetic resonance imaging (MR), positron emission tomography (PET) and computed tomography (CT) have all been well validated to provide detailed information about the cardiac anatomy or the myocardial scar, which is usually the target for substrate-guided ventricular tachycardia ablations [58•, 59, 60, 61]. The current “gold standard” of defining myocardial scar is based on endocardial bipolar voltage recordings. Using a 3D mapping system a roving mapping catheter is moved sequentially along the endocardial surface of the left ventricle. Assuming that the voltage amplitude will be lower on scarred myocardium due to a paucity of live cells, a tiered classification with >1.5 mV for normal myocardium, 0.5-1.5 mV for abnormal myocardium and <0.5 mV for scar is generally accepted for defining scar and its border zone in the left ventricle [62]. These clinical criteria were derived from several animal and patient studies correlating bipolar endocardial voltage recordings to areas of previous myocardial infarction.

Only recently has PET imaging been used to guide and facilitate VT ablation [58•]. Investigators at the University of Maryland showed a good correlation between PET-derived metabolic scar maps and endocardial voltage maps in patients undergoing VT ablation (r = 0.89, p < 0.05). Additionally, 3D scar reconstructions were successfully registered in patients with a commercial mapping system with an acceptable registration error of 3.7 ± 0.7 mm. Scar size, location, and border zone accurately predicted high-resolution voltage map findings (r = 0.87; p < 0.05). After integration of metabolic maps relevant information was available during the procedure. Low voltage recordings within wall segment displaying preserved metabolic activity were shown to be due to suboptimal catheter contact rather than actual myocardial wall disease. Integrated scar maps revealed metabolically active channels within the myocardial scar, which were not detected by voltage mapping. Moreover, PET/CT maps correctly predicted non-transmural epicardial scar that was confirmed with epicardial mapping despite normal endocardial map. Similar results were obtained when using SPECT rather than PET radiopharmaceuticals [59].

An alternate attractive approach is the combination of PET with either CT or MR. While PET provides the metabolic differentiation between normal, hibernating, and scarred myocardium detailed anatomic information can be obtained from CT or MR with a spatial resolution of ≤1 mm or 2–3 mm, respectively. Fusing both datasets can enable a synergistic metabolic and morphological evaluation, which extends beyond what each imaging technique can offer as a stand-alone technology. New elastic algorithms are able to register PET with CT or MR images from separate scanners fast and with an accuracy that is similar to manual elastic registration performed by human experts using up to 32 anatomic landmarks [63].

**Fig. 1:**
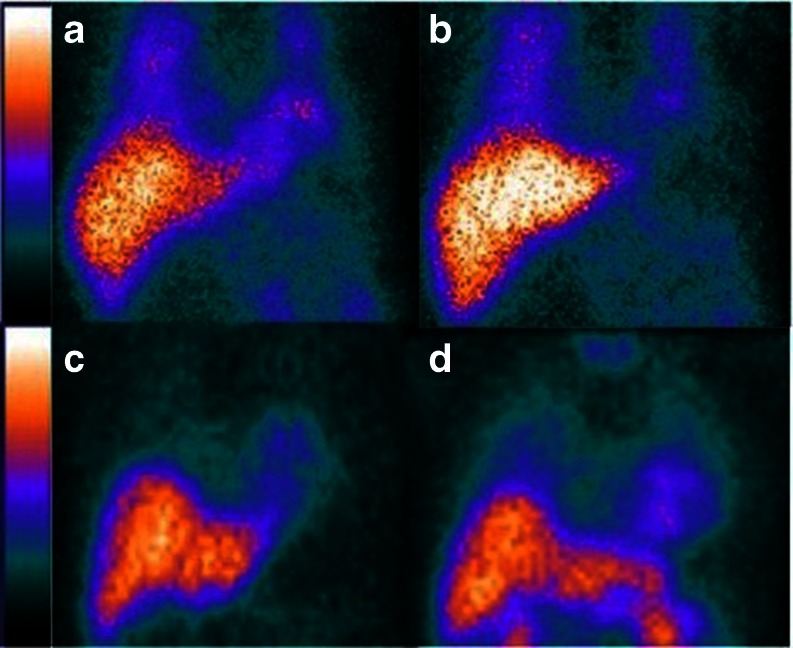
Four-hour delayed MIBG images in panels A and B show decreased uptake of MIBG after ablation in a patient with VT. Panels C and D show increased uptake of MIBG after ablation in a different patient with VT. (A and C: before ablation, B and D: after ablation)

Current ongoing studies evaluate the utility of regional ^123^I-*m*IBG abnormalities to guide ablation in patients with preexisting cardiomyopathy and ventricular arrhythmias. 3D reconstructions of the regional left ventricular ^123^I-*m*IBG innervation have been compared to high density voltage maps. Using the conventional 17-segment analysis [64], the concordance between voltage-defined scar and ^123^I-*m*IBG denervation defect was found to be 75 %. Among the 25 % discordant segments, 20 % of the mismatch segments exhibited a larger^123^I-*m*IBG defect size when compared to the voltage scar. While 90 % of subsequent successful VT ablation sites were found in the area of voltage-defined scar, 10 % were located in an area of abnormal ^123^I-*m*IBG uptake that exhibited preserved voltage [65]. In a subset of patients who underwent repeat ^123^I-*m*IBG imaging within 6 months of VT ablation, there was a trend toward increased late H/M among patients with recurrent VT and decreased late H/M in those without recurrent VT. While this difference did not reach statistical significance, it was hypothesized that regeneration of sympathetic nerves within areas of scar may predispose these patients to VT, and may be reflected by increased ^123^I-*m*IBG (Fig. 1) [66].

## Assessment of Cardiac Innervation with PET Radiotracers

Position emitting radiotracers have also been used to image the cardiac sympathetic nervous system using PET [67, 68]. The most common PET radiotracer studied for this purpose has been carbon-11 labeled hydroxyephedrine (^11^C-HED). ^11^C-HED is taken up by cardiac presynaptic neurons but not metabolized by synaptic degradation enzymes. Similar to the ^123^I-*m*IBG planar and SPECT data, decreased ^11^C-HED PET retention in patients with HF has been associated with increased cardiac mortality and need for cardiac transplantation [69, 70]. PET imaging of the cardiac nervous system is advantageous over single photon imaging due to its superior spacial and temporal resolution compared to planar and SPECT techniques. However, widespread clinical use of ^11^C-HED is limited due to its relatively short 20 minute half-life and complex production requiring an onsite cyclotron, which makes the entire production costly [68].

Reduced cardiac neural regeneration after myocardial infarction has been theorized to be associated with arrhythmia risk. This was tested in a swine model, in which perfusion was assessed by ^13^ N-ammonia and innervation by ^11^C-epinephrine 4 to 12 weeks after myocardial infarction induced by balloon occlusion of the left anterior descending artery. Inducible VT was present in seven of the 11 animals studied, and in those with inducible VT, a significantly larger area of perfusion/innervation mismatch was present [71]. These findings lead to the PARAPET study, a prospective, observational trial, which will assess if hibernating myocardium or inhomogeneity of sympathetic innervation measured with PET can predict sudden cardiac death or cardiovascular mortality [72].

## Conclusions

The clinical and prognostic value of imaging the cardiac nervous system in HF with^123^I-*m*IBG is well established. While the radiotracer has not yet received FDA approval for cardiac application in the US, its potential role may also expand to the arena of electrophysiology, identifying and/or predicting sustained ventricular tachyarrhythmias in patients with cardiomyopathy, determining those who may benefit from ICD implantation, and pinpointing the site of ventricular arrhythmias to guide ventricular tachycardia ablation procedures.
